# Supplementary material for: Factors affecting online health information-seeking behavior in young and middle-aged patients with stroke
Source: PLoS One. 2025 Apr 28;20(4):e0321791. doi: 10.1371/journal.pone.0321791 (PMC12036841; doi:10.1371/journal.pone.0321791)
Supplement: Attachment — Submitted filename: Response to Reviewers.docx [file pone.0321791.s003.docx]

Reviewers' comments:

Reviewer #1:

1.Abbreviations should not be used in the abstract and the methodology should be presented in more detail. Include keywords related to research.

Thanks to the reviewers for their meticulous review and valuable suggestions. Modifications have been made as suggested. In the modification, we removed the abbreviation "OHISB" and fully supplemented it as "online health information seeking behaviour". We supplemented the methodology (lines10-15), including the sampling method, the experimental time period, and the basis for including the dimensions of influencing factors. Keywords were added below the abstract.

2.The introduction is very long and needs to be presented more densely.

Thanks to the reviewers for their meticulous review and valuable suggestions. Modifications have been made as suggested. We have revised the logic and language of the introduction. The data on stroke epidemiology(lines31-33) and online health information seeking behavior(lines45-50) have been replaced with data from other countries that are more representative.

3.The methodology should be presented in more detail. The sample volume formula should be provided. Mention the inclusion and exclusion criteria exactly.

Thanks to the reviewers for their meticulous review and valuable suggestions. Modifications have been made as suggested. We have supplemented the method section in detail, including the sampling method(lines196-199), the sample volume formula(lines199-203), inclusion and exclusion criteria(lines203-209), data collection(lines244-246), and statistical analysis(lines246-250).

4.Discussion emphasizes positive findings and practical implications, while potential negative aspects or challenges are less explored.

Thanks to the reviewers for their meticulous review and valuable suggestions. Modifications have been made as suggested. We have supplemented the negative impacts and current challenges faced in the parts of stroke course(lines368-371), number of combined chronic diseases(lines391-394), and perceived risk(lines407-413).

5.Some references appear to be cited multiple times.There are inconsistencies in the citation style.Ensure proper spacing and punctuation.Improve sentence structure for clarity.

Thanks to the reviewers for their meticulous review and valuable suggestions. Modifications have been made as suggested.We have removed duplicate citations, such as Deng Chaohua(lines146-147),Zhao YC(lines140-141) and Lee(lines456-457).

Additionally, we have checked the full-text citations and sentences.

Reviewer #2:

1.In line 1 abstract, you must state the complete phrase with its abbreviation, and later you can use only the abbreviation. You must write online health information seeking behavior (OHISB) for the first time.

Thanks to the reviewers for their meticulous review and valuable suggestions. Modifications have been made as suggested. In the modification, we removed the abbreviation "OHISB" and fully supplemented it as "online health information seeking behaviour".

2.In introduction section, the statistics used are not up to date. (references 4 and 5)

Thanks to the reviewers for their meticulous review and valuable suggestions. Modifications have been made as suggested. We have updated the data of stroke patients(lines31-33) and young and middle-aged stroke patients(lines34-36).

3.In Theoretical development and hypotheses section, paragraph about “the second Wilson model” has no reference.

Thanks to the reviewers for their meticulous review and valuable suggestions. Modifications have been made as suggested. We have supplemented the references of the second Wilson model, which are Reference 13 and Reference 14 respectively.

4.The methodology section lacks detailed explanations of the data collection and statistical analysis methods. Provide more comprehensive details about the sampling process, data collection instruments, and specific statistical methods used.

Thanks to the reviewers for their meticulous review and valuable suggestions. Modifications have been made as suggested. We have supplemented the content of data collection and statistical analysis methods, including the sampling process(lines196-209), data collection tools(lines244-246) and statistical methods(lines246-250).

5.In discussion section, some sentences haven’t any references for example in Social influence you say: Studies have shown that social influence can significantly improve OHISB in stroke patients. Which studies?Also, in this section, you include more comparisons with previous studies, highlighting how your findings align or contrast with them.

Thanks to the reviewers for their meticulous review and valuable suggestions. Modifications have been made as suggested. We have supplemented the citation of references in the discussion section. Moreover, in the discussion section, we have comprehensively added the comparison between the research results of this study and previous studies, such as in the perception of perceived risk(lines407-418).
