# Supplementary material for: Factors affecting online health information-seeking behavior in young and middle-aged patients with stroke
Source: PLoS One. 2025 Apr 28;20(4):e0321791. doi: 10.1371/journal.pone.0321791 (PMC12036841; doi:10.1371/journal.pone.0321791)
Supplement: Attachment — Submitted filename: Response_to_Reviewers_auresp_2.docx [file pone.0321791.s004.docx]

Dear Editors and Reviewers,

Thank you for your careful review and valuable comments on our manuscript. We have carefully considered each comment and have made the necessary revisions to improve the quality of our manuscript. Below, we provide a detailed response to each of your comments and indicate the changes made in the manuscript.

Reviewers' comments:

Reviewer #1:

1.The article requires significant revisions to improve the language and grammar.

Thanks to the reviewers for their meticulous review and valuable suggestions. We have had the manuscript professionally edited for language by Editageto ensure clarity and adherence to academic writing standards. A certificate of editing has been attached for your reference.

Sincerely,

Ge Shi

Binzhou Medical University

Yaitai,Shandong,China,264000

Email:s18663419862@126.com
